# Barriers and facilitators to pre-exposure prophylaxis uptake among high-risk women and men who have sex with men in Georgia: A pre-implementation mixed-methods study using nominal group technique and the COM-B model

**DOI:** 10.1371/journal.pgph.0006301

**Published:** 2026-06-01

**Authors:** Tamar Mgebrishvili, Irma Kirtadze, David Otiashvili, Mariam Sherozia, David Oliveros, Frederick L. Altice

**Affiliations:** 1 School of Natural Sciences and Medicine, Ilia State University, Tbilisi, Georgia; 2 Addiction Research Center, Alternative Georgia, Tbilisi, Georgia; 3 School of Arts and Sciences, Ilia State University, Tbilisi, Georgia; 4 Section of Infectious Diseases, Department of Internal Medicine, Yale University School of Medicine, New Haven, Connecticut, United States of America; 5 Center for Interdisciplinary Research on AIDS, Yale University School of Medicine, New Haven, Connecticut, United States of America; University of the Witwatersrand Johannesburg Faculty of Health Sciences, SOUTH AFRICA

## Abstract

While pre-exposure prophylaxis (PrEP) research predominantly focuses on men who have sex with men (MSM), PrEP uptake among women who use drugs (WWUD), female sex workers (FSWs), and female partners of people who inject drugs (PWID) is limited. This study fills a critical gap in the literature by examining the unique barriers and facilitators to PrEP uptake among these under-researched female populations, relative to MSM. This pre-implementation study employed a human-centered design approach guided by the Capability, Opportunity, and Motivation-Behavior (COM-B) model. Five focus group sessions using nominal group technique (NGT) were conducted with 66 participants from key populations (MSM, WWUD, FSWs, and female partners of PWID) across three cities (Tbilisi, Kutaisi, and Batumi) to systematically generate and prioritize barriers and facilitators. This study applied the Behavior Change Wheel (BCW) framework to map identified barriers and facilitators to specific intervention functions, highlighting actionable strategies to improve PrEP uptake. Semi-structured interviews with healthcare providers were conducted to contextualize system-level factors. Participants prioritized 15 distinct barriers, with clear differences between groups. MSM most often identified opportunity-related barriers, such as rigid clinic hours, confidentiality concerns, and prior negative healthcare experiences. In contrast, women primarily reported informational and social barriers, including lack of accurate information about PrEP and eligibility, anticipated stigma, and fear of being seen at AIDS centers. Other top barriers included low perceived HIV risk and fear of social stigma. Key facilitators included providing PrEP in neutral, non-stigmatizing delivery settings (i.e., separate from AIDS centers or MSM-specific venues) and offering tele-PrEP and injectable options. Provider insights aligned with participant findings. Findings highlight the need for better access to information and alternative PrEP delivery strategies that decouple PrEP from AIDS centers and MSM-identified venues, including neutral access points, tele-PrEP models, pharmacy or primary care delivery to improve acceptability and reach among at-risk women.

## Introduction

The Eastern European and Central Asian (EECA) region is the largest region globally where new HIV infections are growing fastest [1]. Nearly all (94%) new HIV cases in EECA are concentrated in key populations, including men who have sex with men (MSM) and people who inject drugs (PWID), and their sexual partners [2]. With these two groups driving the epidemic, there were an estimated 140,000 people with HIV (PWH) in EECA in 2023, with annual incidence increasing by 33% since 2010 [2]. New HIV infections, however, are preventable.

The HIV epidemic in Georgia remains a significant public health challenge. The country has a concentrated epidemic with an estimated adult HIV prevalence of 0.4%. Prevalence is highest among MSM (15.5%) [3], and remains relatively low among female sex workers (FSWs) (0.3%) [4] and PWID (0.9%) [5]. While injecting drug use has historically been the leading cause of HIV, heterosexual transmission has recently surpassed it. In 2012, heterosexual contact accounted for 45% of newly registered HIV cases. This figure increased to 53% in 2024, suggesting a growing number of infections among partners of PWID and a feminization of the epidemic [6]. Despite PWID and MSM being the highest-risk groups, results of epidemiological studies suggest that only 30.4% of PWID and 48.4% of MSM are engaged in HIV prevention programs, and fewer than half know their HIV status [3,5,7]. Late identification remains a challenge, with 55% of new cases of HIV diagnosed late and 34% in a very advanced stage.

Systematic reviews and meta-analyses demonstrate that PrEP is highly effective for preventing HIV acquisition across multiple high-risk populations. Despite its safety and efficacy, consistent evidence shows that low awareness, limited knowledge, and structural barriers to access remain major rate-limiting factors for PrEP uptake and implementation [8–10]. Other significant hurdles include low self-perceived risk, concerns about side effects, dosing modalities (daily vs. on-demand), stigma, and competing life events [11,12]. Furthermore, linkage from HIV testing to a PrEP prescription occurs for only 21–67% of those referred. Even when PrEP is free and widely available, numerous barriers often persist at the patient, clinician, or healthcare system levels.

In 2017, Georgia became the first country in Eastern Europe to introduce PrEP services. Initially targeting MSM and transgender (TG) populations, national guidelines expanded eligibility in 2022 to include sero-discordant couples, PWID, and FSWs. PrEP is delivered primarily by infectious disease specialists at the National AIDS Center in partnership with a large LGBTQ+ community-based organization (CBO). While PrEP was initiated in 2019 at the CBO and on-demand PrEP was supported in 2023, geographic access remains concentrated in Tbilisi, with recent expansion to Batumi and Kutaisi [13]. Recruitment is largely driven by incentivized peer workers, involving onsite screening for HIV/STIs followed by accompanied referral to the AIDS Center. A newly introduced initiative enables MSM to access PrEP and HIV screening services at community centers twice a week, through visits by infectious disease specialists from the AIDS center. According to the national protocol covering all high-risk groups, PrEP is delivered using two regimens: a daily oral pill of tenofovir disoproxil fumarate/emtricitabine (TDF/FTC) available to all high-risk populations, and an on-demand regimen (TDF/FTC, “2-1-1”) offered only to MSM [14].

Despite these efforts, uptake remains uneven. As of 2024, only 2,054 people in Georgia had received PrEP at least once [6]. Although PWID are eligible under national guidelines, PrEP initiation has not been reported among individuals recorded as PWID in existing data systems. Furthermore, the first cases of women initiating PrEP emerged only in 2023, with a total of 26 women initiating treatment since then (18 in Sero-discordant couples and 8 FSWs) [6]. Although the National HIV/AIDS Strategy 2023–2025 identifies an urgent need to expand PrEP access to FSWs, PWID, and their partners [13], current PrEP delivery in Georgia remains largely centralized and oriented toward MSM-focused service delivery models, which may not adequately address the needs of other key populations, particularly women. Structural and service-level barriers, including reliance on AIDS centers and limited integration into broader healthcare settings, may contribute to low uptake among underrepresented groups. A pre-implementation, human-centered approach is therefore needed to better understand population-specific barriers and inform more accessible and acceptable PrEP delivery strategies.

While global PrEP research has largely focused on MSM [15,16], evidence on barriers and facilitators among women who use drugs (WWUD) and FSWs remains limited [17–20]. Existing studies suggest that WWUD and FSWs face distinct barriers to PrEP uptake, including concerns about stigma, competing daily priorities, low perceived relevance, and preferences for delivery models that minimize clinic-based visibility. Recent work has also highlighted interest in long-acting injectable PrEP among women, particularly where daily pill-taking is viewed as burdensome or difficult to conceal [17–20]. These findings, however, come largely from settings outside Eastern Europe and may not translate directly to the Georgian context. Besides, female partners of PWID are a critical group for PrEP research because heterosexual transmission now contributes to an increasing proportion of new HIV infections in Georgia, reflecting the feminization of the epidemic. These women may face unique social and structural vulnerabilities, and potential exposure through their partners’ injecting practices, which are not captured when studying PWID alone. Including this group allows for a more comprehensive understanding of barriers and facilitators among high-risk women.

This study addresses this gap by examining under-researched female populations compared with MSM in Georgia, providing insights to guide tailored interventions. Consequently, this research aims to examine the barriers and facilitators to PrEP uptake among high-risk groups in Georgia, including WWUD (recent or current drug use), female partners of male PWID, MSM, and FSWs. Specifically, the study aims to identify and prioritize key barriers and facilitators and to compare these factors across population groups.

## Materials and methods

### Ethics statement

The study was approved by the Institutional Review Boards (IRB) of the Health Research Union (00009520) and Yale University (2000038594). Participants read an IRB-approved consent script and provided verbal informed consent prior to participation. Verbal consent was documented through audio-recording of each participant’s agreement, and a member of the research team witnessed the consent process. NGT participants were paid (15 USD equivalent) for their time and transportation costs.

### Study design and framework

This pre-implementation study utilized a human-centered design approach to identify barriers and facilitators to PrEP uptake. The study was guided by the Capability, Opportunity, Motivation-Behavior (COM-B) model [21], which maps individual-level determinants of behavior. We employed a convergent mixed-methods design, combining qualitative data generation with quantitative prioritization of participant-identified barriers using NGT [22,23] to systematically generate and prioritize barriers and facilitators, followed by qualitative mapping of these factors to COM-B domains to inform potential interventions via the Behavior Change Wheel (BCW) [21]. NGT has been used extensively in pre-implementation research [24–29] and was utilized primarily to rapidly elicit and prioritize perceived barriers to PrEP uptake. Facilitators were discussed qualitatively but not rank-ordered, given low baseline familiarity with PrEP among several participant groups. Integration of qualitative and quantitative components occurred at the interpretation stage, where prioritized barriers and qualitatively identified facilitators were triangulated across participant groups and provider interviews.

### Setting and participants

Participants were recruited via purposive sampling from community organizations in Tbilisi, Kutaisi, and Batumi serving MSM, PWID, and FSWs. Community organizations were selected based on their established engagement with key populations, geographic coverage across the three largest urban centers, and their role in HIV prevention and harm reduction service delivery. Participants were recruited and enrolled between May 17, 2025 and June 14, 2025. Recruitment was facilitated by staff from service provider organizations who screened for eligibility: (1) member of a high-risk group (MSM, WWUD, FSW, or female partner of a PWID); (2) age ≥ 18 years; (3) self-reported HIV-negative status; and (4) Georgian language proficiency. The number of NGT sessions per population group was determined to ensure representation across key populations while maintaining feasibility within the study timeline. Multiple groups were conducted to capture a range of perspectives and enhance the robustness of prioritized findings.

To contextualize participant-identified barriers, we conducted semi-structured interviews with four service delivery providers, including 2 infectious disease physicians and 2 harm reduction staff involved in HIV prevention or PrEP referral. Interviews explored PrEP referral pathways, confidentiality concerns, protocol constraints, inter-organizational coordination, and perceptions of stigma.

### Data collection

Data were collected through single-gender NGT sessions (avg. 8 participants, range: 5–11) conducted at community centers in Georgian language. The sessions followed a standard four-stage procedure: 1) Silent generation of ideas regarding barriers and facilitators to PrEP; 2) Round-robin recording of ideas without debate; 3) Group discussion for clarification of ideas; and 4) Voting, where participants were given three votes to allocate across items they deemed most important; votes could be distributed across multiple items (e.g., one vote each to three items), split unevenly (e.g., two votes to one item and one to another), or concentrated on a single item (all three votes). Unweighted votes were flexibly allocated, meaning participants could assign all three votes to a single idea or split three votes to three different ideas, according to their preferences. While barriers to PrEP uptake were explored using the full NGT process, including participant voting to prioritize items, facilitators were explored only qualitatively. This is because identifying facilitators often required participants to imagine strategies that could improve PrEP uptake. Given that many participants had limited prior knowledge or experience with PrEP, they were unable to meaningfully rank facilitators. Sessions were audio-recorded and transcribed verbatim. Semi-structured interviews with healthcare providers lasted 45–60 minutes. NGTs and interviews were conducted in Georgian and later transcribed and translated into English for analysis.

### Data analysis

Barriers identified during the NGT sessions were prioritized through independent voting, with votes summed and ranked to determine the most important barriers for each key population. For consistency with the barriers analysis and to facilitate comparison across COM-B domains, facilitators were presented in a structured table indicating which groups mentioned each item and the number of groups citing it, providing an indication of how broadly each facilitator was mentioned across groups. NGT discussions were transcribed verbatim and analyzed using reflexive thematic analysis [30]. The coding structure included three levels: (1) codes derived from participant statements, (2) sub-themes organizing related codes, and (3) overarching COM-B domains. Themes were compared across MSM, FSW, and WWUD groups to identify shared and unique barriers and facilitators. Coding was conducted manually in Microsoft Excel to enable direct integration of transcript data with the structured NGT outputs, including participant-generated items, rankings, and thematic categories. Given the relatively small and highly structured dataset, Excel was well suited for transparent organization, comparison across groups, and linkage of qualitative findings to ranked items. Two independent coders (TM and DOt) reviewed all transcripts and applied the coding framework iteratively. They met regularly to compare coding decisions, resolve discrepancies through consensus, and refine interpretation where needed. Analytic memos were used throughout to document coding decisions, areas of disagreement, and the rationale for final thematic assignments. This process enhanced transparency, consistency, and the auditability of mapping themes to COM-B domains.

The structured NGT format supported transparent organization and prioritization of participant-generated ideas. After inductive theme development, themes were mapped deductively to the COM-B framework [21] to identify relevant behavioral determinants. Analysis focused on psychological capability, social/physical opportunity, and reflective motivation; domains not represented in the data (physical capability, automatic motivation) were excluded. Finally, identified determinants were mapped to intervention functions using the BCW to link findings to actionable strategies.

Provider interviews were analyzed using a reflexive thematic approach. Coding was conducted by trained qualitative researchers familiar with the Georgian HIV prevention context. Themes were developed inductively form the data, with analytic memos used to document interpretations and coding decisions. Codes were then mapped deductively to COM-B domains through team discussion to minimize undue framework imposition. Discrepancies were resolved through consensus. Analysis focused on themes related to PrEP service delivery, referral pathways, and perceived barriers to uptake. Findings from provider interviews were used to contextualize and triangulate participant-identified barriers and facilitators, highlighting areas of convergence and divergence between provider perspectives and participant lived experiences.

### Reflexivity statement

Several members of the research team are Georgian and have prior experience working in HIV prevention programs. This insider perspective facilitated recruitment and understanding of local HIV prevention contexts but may have shaped interpretation of participants’ responses. To address this, coding and thematic analysis were conducted collaboratively with team members from outside the immediate context, and analytic memos were used to document decisions and reflect on potential biases.

## Results

### Participant characteristics

A total of 66 participants (16 males, 50 females) were enrolled across five focus groups representing key high-risk populations: MSM currently on PrEP, MSM who discontinued PrEP, WWUD, FSWs, and female partners of PWID. The mean age of participants was 36 years (range 18–63). Notably, no female participants had ever initiated PrEP. Additionally, four service providers (two men, two women) were interviewed to contextualize systemic barriers. Detailed demographic characteristics of participants in the NGT discussions are presented in Table 1.

**Table 1:** Demographic and PrEP-related characteristics of study participants, stratified by high-risk groups.

High-risk groups	n	Mean Age (SD, range)	PrEP Status (n)
**WWUD**	28	34 (12, 22-55)	Never been on PrEP
**Female partners of PWID**	10	48 (11, 26-63)	Never been on PrEP
**MSM with PrEP experience**	16	26 (6, 18-42)	7 currently using; 9 discontinued
**FSW**	12	46 (9, 33-58)	Never been on PrEP
**Total**	66	36 (13,18-63)	16

**Legend:** WWUD = Women Who Use Drugs, PWID = People Who Inject Drugs, MSM = Men Who Have Sex With Men, FSW = Female Sex Workers.

### Rank-ordered barriers to PrEP uptake

Participants generated 23 distinct barriers, of which 15 were prioritized through voting. The cumulative voting results, stratified by risk group, are detailed in Table 2. The highest-ranked barriers overall were limited awareness of PrEP (n = 45 votes), stigma/self-stigma regarding clinic attendance (n = 37), low perceived HIV risk (n = 27), limited awareness of HIV/STI risks (n = 23), and concerns about side effects (n = 14).

**Table 2:** Prioritization of barriers to PrEP in Georgia, stratified by COM-B domains (n = 5 groups).

Participant-generated barriers to PrEP uptake	COM-B Domain	MSM that discontinued PrEP (n = 7)	MSM on PrEP (n = 9)	WWUD (n = 28)	FSW (n = 12)	Female partners of PWID (n = 10)	Total Votes (n = 198)	Final Rank
Limited awareness of PrEP	Capability	4	0	28	5	8	45	1
Stigma/self-stigma visiting clinics	Opportunity	0	1	18	0	18	37	2
Low perceived HIV risk/ “no need”	Motivation	1	4	3	17	2	27	3
Limited awareness of HIV/STI risks	Capability	0	0	17	6	0	23	4
Perceived side effects/ interactions	Capability	2	3	9	0	0	14	5
Lack of accurate info (PrEP only for gay men)	Capability	4	6	2	0	0	12	6
Lack of trust/confidentiality concerns	Opportunity	1	5	4	1	0	11	7
Daily PrEP not comfortable	Capability	4	3	1	0	0	8	8
Laziness/ not caring for own health	Motivation	0	0	2	3	2	7	9
Rigid clinic hours/ hard-to-reach	Opportunity	1	4	0	0	0	5	10
Having permanent partners	Motivation	0	0	0	4	0	4	11
PrEP stigmatized as linked to promiscuity	Motivation	2	0	0	0	0	2	12
National protocol not user-friendly	Opportunity	0	1	0	0	0	1	13
Lack of delivery services	Opportunity	1	0	0	0	0	1	14
Drug resistance risk	Motivation	1	0	0	0	0	1	15

**Legend:** WWUD = Women Who Use Drugs, PWID = People Who Inject Drugs, MSM = Men Who Have Sex With Men, FSW = Female Sex Workers.

### Qualitative findings by COM-B domain

Although several barriers were shared across female groups, WWUD and female partners of PWID most strongly emphasized lack of PrEP awareness and stigma associated with AIDS-centered services, whereas FSWs more often emphasized low perceived need for PrEP in the context of routine condom use. The quantitative rankings were contextualized through qualitative thematic analysis, mapped to the COM-B domains of Capability, Opportunity, and Motivation.

1. *Psychological Capability: Awareness and Knowledge*

Limited Awareness: Lack of accurate information was classified primarily as a Capability barrier, but participants’ narratives indicated that this misconception was reinforced by Opportunity constraints, specifically PrEP’s delivery through MSM-identified services. Lack of PrEP awareness was most pronounced among WWUD and female partners of PWID, with the majority of women overall (33 out of 50) had never heard of PrEP prior to the study. A pervasive misconception was that PrEP is exclusively for MSM. As one WWUD participant noted:

*“I thought it was only for gay men. Once I tried to order a box from the community center (here: center that serve to MSM only) with preventive supplies like condoms and lubricants, and it said it was only for MSM. So, I automatically assumed that, since they also deliver PrEP, it wouldn’t be for me.”*

Risk Perception: While FSWs and MSM demonstrated slightly higher awareness, FSWs frequently perceived themselves at low risk due to consistent condom use. Some conflated HIV/STI detection with prevention, believing that regular condom use or testing alone was sufficient to reduce their personal risk. As one FSW explained:

*“I use condoms with every client, and sometimes even multiple condoms at once to avoid tearing... I prevent the spread of infections... I am afraid that if I spread infections, someone could harm me”.*

This statement illustrates how reliance on condoms can contribute to a perception of low personal HIV risk, which in turn may reduce motivation to initiate PrEP. This aligned with provider observations that FSWs often view Hepatitis C as a more immediate threat than HIV. Some providers also felt that PrEP may not be strictly necessary for these groups, given the low perceived HIV prevalence, partially echoing participants’ own perception of low PrEP need. Providers also indicated that condom use is high among FSWs, and many of them believe that this alone is sufficient for HIV prevention, largely aligning with participants’ perception of low PrEP necessity.

Side Effects: Concerns about interactions with alcohol, street drugs, or opioid agonist therapy (methadone/buprenorphine) were prominent among WWUD.

2. *Social and Physical Opportunity: Access and Stigma*

Stigma: Stigma associated with visiting HIV/AIDS centers was the second highest-ranked barrier. Participants feared that being seen at these clinics signaled an HIV-positive status or “deviant” behavior. An MSM participant shared:

*“My family found [my PrEP]... and immediately assumed I was gay... So, I stopped taking PrEP”.*

Shame and societal pressure around visiting a doctor for PrEP was the highest ranked among WWUD and female partners of PWID. They also noted fear of judgment or poor treatment by healthcare providers. Some had experienced or worried about discrimination when discussing PrEP or sexual behavior. Internalized stigma (shame about seeking PrEP) was mentioned by WWUD as well. One WWUD participant noted:

*“In every field, doctors treat us poorly…a doctor couldn’t find my veins, and he made a huge scene because I was a drug user.”*

Because PrEP is delivered exclusively through AIDS centers or MSM-focused community organizations, visiting these locations carried symbolic meaning that influenced participants’ decision-making. Being seen at these facilities was interpreted as a sign of sexual orientation or drug use, creating a strong cognitive disincentive to access PrEP, especially in regions, outside the capital. This reflects anticipated stigma and confidentiality concerns that constrained perceived opportunity to access PrEP services.

Access: Rigid clinic hours (10:00–16:00) and the remote location of the AIDS Center were major logistical hurdles, particularly for employed MSM. One participant described the travel requirement as a “disaster,” necessitating time off work.

Service Gaps: Providers highlighted a structural inequity in service delivery. While MSM can access PrEP through community-based organizations with integrated support, comparable outreach and service infrastructure is lacking for WWUD and FSWs. Female partners of PWID were particularly underserved, as they are not directly engaged through either MSM-focused or harm reduction programs. A harm reduction provider emphasized:

*“Our beneficiaries are not the type who would go to an AIDS center or an MSM-focused community to get PrEP”.*

This challenge is compounded by the fact that, unlike staff in MSM-focused centers, harm reduction workers serving PWUD are limited to educational duties and do not receive financial compensation for broader PrEP-related support.

3. *Reflective Motivation: Trust and Privacy*

Trust and confidentiality were critical drivers of motivation. For MSM, the lack of anonymity in specialized clinics was a deterrent. Some described using disguises (sunglasses, hats) to visit clinics. Conversely, older WWUD with experience in HCV treatment programs were less concerned about data privacy, whereas younger feared inclusion in state registries.

### Facilitators and intervention mapping

Due to low baseline knowledge of PrEP among several participant groups, facilitators could not be rank-ordered. Participants, however, qualitatively identified several strategies to improve PrEP uptake (summarized in Table 3). Facilitators clustered around three broad themes: enhancing knowledge and awareness, improving access and delivery, and reducing stigma through inclusive and flexible approaches. In contrast to barriers, limited heterogeneity was observed across female participant groups with respect to facilitators, with WWUD, FSWs, and female partners of PWID identifying largely similar strategies to improve PrEP uptake.

**Table 3:** Summary of facilitators to expand PrEP in Georgia, stratified by COM-B domains (n = 5 groups).

Participant-generated facilitators to PrEP uptake	COM-B Domain	MSM on PrEP	MSM that discontinued PrEP	WWUD	FSW	Female partners of PWID	Total Groups Mentioned
Increased awareness and education about PrEP	Capability	X	X	X	X	X	5
Routine discussion and offering of PrEP by healthcare providers	Capability	X	X	X	X	X	5
Correct marketing to include women and general population (not only MSM)	Opportunity	X	X	X	X	X	5
Easily accessible information in Georgian	Capability	X	X	X	X	X	5
Expansion of PrEP access points and neutral delivery settings	Opportunity	X	X	X	X	X	5
Offering injectable PrEP for those who prefer it over daily oral regimen	Opportunity	X	X	X	X	X	5
Outreach adapted to online FSWs and hard-to-reach populations	Opportunity			X	X	X	3
Flexible scheduling and ability to reserve appointments	Opportunity		X	X			2
Integration of PrEP into community centers serving WWUD	Opportunity			X		X	2
Information about PrEP interactions with substances and alcohol	Capability			X			1

**Legend:** WWUD = Women Who Use Drugs, PWID = People Who Inject Drugs, MSM = Men Who Have Sex With Men, FSW = Female Sex Workers.

1. *Psychological capability: Enhancing Knowledge and Awareness*

Participants across all groups emphasized the need for clear, accessible information about PrEP and its use. This included increasing awareness of PrEP in the general population, providing information in Georgian, and routine discussions by healthcare providers. One FSW noted that outreach through online platforms and peer networks would be particularly helpful for reaching women not engaged with traditional health services.

2. *Physical/social opportunity: Improving Access and Delivery*

Facilitators highlighted the importance of expanding PrEP access points and offering neutral, non-stigmatizing delivery settings. MSM, female participants and service providers alike suggested integrating PrEP into community centers, primary care, and gynecology clinics to reduce the association with AIDS centers or MSM-focused venues. As one MSM-focused service provider mentioned:

*“There are some subgroups of MSM who are hard to reach by our social workers. They do not want to be affiliated with community centers. I know people who want to take PrEP but they prefer more neutral settings, such as pharmacies. In the regions, some individuals do not even want to walk past an MSM-focused community organization, let alone go there to get PrEP.”*

Flexible scheduling, the ability to pre-book appointments, and tele-PrEP were suggested to accommodate participants’ daily responsibilities and reduce logistical barriers. Several participants expressed interest in long-acting injectable PrEP as an alternative to daily oral regimens, citing convenience and privacy considerations.

3. *Reflective Motivation: Reducing Stigma through Inclusive and Flexible Approaches*

Participants emphasized the need for inclusive marketing campaigns that explicitly target women and other high-risk populations, countering the perception that PrEP is only for MSM.

*“When marketing presents information about PrEP, it simultaneously emphasizes that it’s for gay people. This needs to change. It should also be marketed in a way that includes and appeals to women.”*

Privacy protections, discreet packaging, and telehealth options were recommended to address concerns about stigma and confidentiality. For example, an MSM participant described stress caused by noisy pill bottles revealing his medication status while traveling, highlighting how seemingly minor design issues can affect acceptability.

Finally, identified barriers and facilitators were mapped to the Behavior Change Wheel (BCW) to propose actionable interventions. Table 4 links these findings to specific intervention functions and policy levers, such as expanding PrEP delivery points, implementing mass media campaigns with neutral messaging, and providing training for healthcare providers to routinely offer PrEP information to diverse populations. This integrated approach demonstrates how facilitators, even without ranked prioritization, can inform actionable strategies for improving PrEP uptake among high-risk groups.

**Table 4:** COM-B domains and behavior change wheel intervention functions for barriers and facilitators of PrEP uptake in high-risk groups in Georgia.

COM-B Domain	Key Barrier	Suggested Intervention Function(s)	Policy/Implementation Levers
Psychological Capability	Limited awareness of PrEP	Education, Training	Community workshops, mass media campaigns, educational materials in Georgian
	Limited awareness of HIV/STI risks	Education, Training	Integration of PrEP information into routine consultations; provider training
	Concerns about side effects/ interactions	Education, Persuasion	Counseling about PrEP interactions with substances and medications
	Misperception that PrEP is only for MSM	Education, Persuasion	Inclusive messaging targeting women and general population
Physical/Social Opportunity	Stigma/ self-stigma visiting clinics	Environmental restructuring, Modeling, Persuasion	Neutral delivery points, tele-PrEP, privacy protections
	Lack of trust/ confidentiality concerns	Enablement, Environmental restructuring	Discreet packaging, tele-PrEP, anonymous service options
	Rigid clinic hours/ hard-to-reach locations	Environmental restructuring, Enablement	Flexible hours, mobile clinics, appointment windows
	Limited delivery points/ centralized access	Environmental restructuring, Enablement	Integration into primary care, pharmacies, harm reduction and community centers for WWUD/FSW
Reflective Motivation	Low perceived HIV risk/ “no need”	Education, Persuasion	Risk perception campaigns, counseling sessions
	Laziness/ not caring for own health	Persuasion, Incentivization	Reminder apps, adherence support interventions
	PrEP stigmatized as linked to promiscuity	Persuasion, Modeling	Normalize PrEP via testimonials, social media campaigns

**Legend:** WWUD = Women Who Use Drugs, PWID = People Who Inject Drugs, MSM = Men Who Have Sex With Men, FSW = Female Sex Workers.

## Discussion

### Filling the gap: PrEP beyond MSM

While the global body of literature on PrEP barriers is extensive, it remains overwhelmingly concentrated on MSM. Research addressing the unique needs of women at high risk - particularly WWUD and female partners of PWID - remains scarce, both globally and specifically within the Eastern European context. This study addresses this critical gap by providing the first in-depth examination of PrEP barriers among these high-risk female populations in Georgia. By expanding the lens beyond the LGBTQ+ community, our findings reveal a stark divergence in the prevention landscape: while the epidemic in Georgia is increasingly driven by heterosexual transmission, the prevention infrastructure, specifically PrEP, remains largely siloed around MSM, leaving high-risk women poorly reached by the current prevention infrastructure.

### The knowledge gap: A unique challenge

The most striking finding of this study is the very limited PrEP awareness among women in our sample, particularly among WWUD and female partners of PWID. This stands in sharp contrast to global trends among MSM, where systematic reviews indicate awareness levels of up to 70%, even if uptake varies [15,16]. Our findings, however, align more closely with recent data from sub-Saharan Africa, where only about 14% of high-risk women demonstrated adequate PrEP awareness [31], suggesting that without targeted intervention, awareness among women does not naturally follow the trajectory of MSM. In our study, the pervasive misconception that PrEP is a “gay medication” appears to be a direct consequence of Georgia’s siloed implementation history. Unlike in settings where PrEP is successfully marketed to youth and women via peer-led social media campaigns [32], Georgian prevention messages have unintentionally reinforced a perception that the intervention is “not for them.” The perception of PrEP as a “gay medication” in Georgia appears to be rooted in both structural and historical factors. PrEP services were initially rolled out exclusively through MSM-focused community organizations and the National AIDS Center, with eligibility and outreach largely targeting MSM and transgender populations. Marketing campaigns, peer outreach, and educational materials similarly concentrated on LGBTQ+ networks, inadvertently signaling to women and other populations that PrEP was not intended for them. This framing likely reinforced misconceptions and limited engagement among high-risk women, including WWUD, FSWs, and female partners of PWID.

### Stigma and the “MSM-Only” ecosystem

Social opportunity was severely constrained by the specific setting of service delivery. Our findings extend the literature on anticipated stigma by showing that for WWUD and female partners of PWID, visiting an MSM-linked community center or the national AIDS Center is not just inconvenient but it is stigmatizing. This mirrors findings from the United States, where MSM in rural areas often avoid community clinics due to the fear of being “outed” by association [33,34]. Women in this context described active risk-management strategies, including condom use and testing; however, PrEP was not integrated into these strategies, suggesting a gap in how PrEP is framed relative to women’s existing prevention logic rather than an absence of risk awareness. This is consistent with recent findings from Iran, where WWUD and FSWs cited the fear of disclosing risky behaviors in clinical settings as a primary deterrent to PrEP access [35]. This convergence of international evidence suggests that specialized “high-risk” clinics, while effective for initially attracting some users, eventually become barriers for hidden populations who prioritize anonymity.

### Service delivery: The need for neutrality and flexibility

Stigma emerged as a behavioral mechanism operating through anticipated disclosure, symbolic association of AIDS centers with HIV infection, and fear of social visibility. These factors reduced perceived opportunity to seek PrEP and weakened reflective motivation, even among participants who recognized HIV risk. To bridge this gap, service delivery must shift from specialized to “neutral” settings. Our participants’ strong preference for pharmacy-based delivery is supported by a growing body of evidence from the U.S. and Kenya, which demonstrates that community pharmacies can effectively reduce stigma and improve access for hard-to-reach groups, provided that staff receive privacy training [36,37]. Integrating PrEP into routine gynecology and primary care would further normalize the intervention, dissociating it from sexual identity or drug use.

While participants emphasized the need for neutral delivery settings to reduce stigma and reach women, it is important to recognize that MSM-focused community organizations have been foundational in establishing PrEP services in Georgia for MSM. These organizations possess established trust, peer networks, and specialized knowledge that are critical for engaging MSM and other key populations. Expanding PrEP delivery to neutral or primary care settings should therefore be pursued in parallel with, rather than at the expense of, existing MSM-focused services. Careful coordination is required to avoid disrupting current provision while ensuring that new service models effectively engage WWUD, FSWs, and other hard-to-reach groups. Pilot programs and stakeholder consultations could help design complementary approaches that leverage existing strengths while addressing gaps in access and confidentiality.

Barriers related to physical opportunity (rigid hours, daily dosing burdens) necessitate flexible delivery models. Consistent with studies involving women who engage in sex work and drug use in the U.S., our participants identified injectable PrEP as a critical facilitator to overcome the “burden” of daily pill-taking [17–20]. Currently, long-acting injectable options, such as lenacapavir and cabotegravir, are not available in Georgia and would require local regulatory approval and access channels. While global agreements allow generic production for 120 high-incidence, resource-limited countries, it is not yet clear whether Georgia is included in the initial rollout. If introduced, injectable PrEP could help address barriers such as stigma related to clinic visits, rigid clinic hours, and adherence challenges, but implementation would require careful planning around regulatory approval, pricing, distribution, provider training, and integration into existing prevention services. Additionally, the clear demand for Tele-PrEP and appointment scheduling apps in our study echoes the success of digital health interventions for young MSM of color in other settings, where apps provided essential confidentiality and provider linkage [38,39].

### Policy and implementation levers

Participants’ priorities point to implementation strategies focused on improving acceptability, appropriateness, and reach. These include differentiated care models such as delivering PrEP through neutral or primary care settings, pharmacy-based refills, tele-PrEP models that reduce clinic visibility, and clearer role delineation between harm reduction organizations and prescribers to streamline referral pathways. Further, translating these findings into practice requires specific policy shifts. First, national protocols must be flexible enough to accommodate on-demand (2-1-1) PrEP users (primarily MSM), removing the burden of regular three-month visits for those with infrequent risk. Second, funding mechanisms must expand beyond MSM-specific channels. The current lack of incentives for harm reduction providers to discuss PrEP creates a missed opportunity; as noted in similar studies of “bridge clinics” for WWUD, these providers already possess the requisite trust but lack the mandate to integrate PrEP [20]. Finally, provider training on confidentiality is essential. The fear of state registries expressed by younger WWUD highlights that trust is a fragile component of reflective motivation; ensuring absolute data privacy is a prerequisite for engaging this demographic [40].

While this study was conducted in major urban centers (Tbilisi, Batumi, and Kutaisi), the findings have important implications for rural areas in Georgia, where access to HIV prevention services is more limited and stigma may be more pronounced. In such settings, scalable service delivery models, including tele-PrEP, pharmacy-based provision, and integration of PrEP into primary care, may help extend reach beyond specialized urban clinics. However, successful implementation in rural contexts will require adaptation to local infrastructure, workforce capacity, and community norms, as well as targeted outreach to populations not currently engaged in prevention services.

### Limitations

Despite the many new and important findings, this study is not without limitations. First, although NGT minimizes dominance effects through private voting, which is a key strength in NGT through piggy-backing of ideas, group dynamics may still influence idea generation. Second, while barriers were systematically prioritized using the NGT voting process, facilitators were explored qualitatively and were not rank-ordered because of participants’ limited baseline familiarity with PrEP. Accordingly, barrier findings reflect participant-prioritized concerns, whereas facilitator findings reflect descriptively identified themes. This asymmetry limits direct comparison between barriers and facilitators, and facilitator findings should be interpreted as exploratory rather than as measures of relative priority across groups. Third, recruitment via community centers means our sample was already engaged in some form of service. As a result, awareness, attitudes, and reported barriers may be disproportionally represented compared to populations not currently accessing such services. This suggests that knowledge gaps, stigma, and logistical challenges could be even more pronounced among harder-to-reach individuals, particularly women who are not connected to HIV prevention programs. Consequently, caution is warranted when generalizing these findings to all high-risk populations in Georgia. Fourth, while our sample covers major urban areas (Tbilisi, Batumi, Kutaisi), findings may not be fully transferable to rural Georgia. The study focused primarily on individual-level barriers and facilitators**,** and certain COM-B domains, including automatic motivation and physical capability**,** could not be fully explored. Although we present demographic characteristics of participants, data on education, employment, and duration of engagement with HIV prevention services were not collected. Finally, the NGT was conducted in Georgian, limiting the inclusion of migrant populations or non-Georgian speakers. Notwithstanding these constraints, this study represents the first systematic investigation of PrEP barriers among these specific high-risk populations in Georgia. By centering the perspectives of historically under-researched groups our research provides unique and actionable insights for the implementation of tailored HIV prevention services.

## Conclusions

This pre-implementation study demonstrates that PrEP scale-up strategies centered on AIDS centers and MSM-focused organizations can unintentionally reproduce gendered inequities in PrEP access. Addressing anticipated stigma, improving confidentiality, and reframing PrEP delivery through neutral and flexible service models (e.g., tele-PrEP, extended clinic hours, wider appointment windows, and, when available, multiple PrEP formulations including long-acting injectables), are critical to expanding PrEP reach among women at risk. Implementation strategies that align with women’s existing prevention practices may enhance equitable PrEP uptake. Future work should focus on pilot testing these proposed delivery models and informing national policy revisions to ensure that PrEP services are both inclusive and accessible to all high-risk populations in Georgia.
